# Behavior out of control: Experimental evolution of resistance to host manipulation

**DOI:** 10.1002/ece3.5294

**Published:** 2019-06-02

**Authors:** Nina Hafer‐Hahmann

**Affiliations:** ^1^ Department of Evolutionary Ecology Max Planck Institute for Evolutionary Biology Plön Germany; ^2^ EAWAG Swiss Federal Institute of Aquatic Science and Technology Dübendorf Switzerland

**Keywords:** copepod, host manipulation, *Macrocyclops albidus*, resistance, response to selection, *Schistocephalus solidus*

## Abstract

Many parasites alter their host's phenotype in a manner that enhances their own fitness beyond the benefits they would gain from normal exploitation. Such host manipulation is rarely consistent with the host's best interests resulting in suboptimal and often fatal behavior from the host's perspective. In this case, hosts should evolve resistance to host manipulation. The cestode *Schistocephalus solidus* manipulates the behavior of its first intermediate copepod host to reduce its predation susceptibility and avoid fatal premature predation before the parasite is ready for transmission to its subsequent host. Thereafter, *S. solidus* increases host activity to facilitate transmission. If successful, this host manipulation is necessarily fatal for the host. I selected the copepod *Macrocyclops albidus*, a first intermediate host of *S. solidus,* for resistance or susceptibility to host manipulation to investigate their evolvability. Selection on the host indeed increased host manipulation in susceptible and reduced host manipulation in resistant selection lines. Interestingly, this seemed to be at least partly due to changes in the baseline levels of the modified trait (activity) rather than actual changes in resistance or susceptibility to host manipulation. Hence, hosts seem restricted in how rapidly and efficiently they can evolve resistance to host manipulation.

## 1 INTRODUCTION

Host manipulation is the alteration of a host's phenotype, such as its behavior, by a parasite that increases parasite fitness beyond benefits the parasite would gain from normal exploitation. Some of the most intriguing examples of host manipulation come from trophically transmitted parasites that alter host behavior or induce other phenotypic changes that render their host more prone to predation (Holmes & Bethel, 1972; Moore, 1984, 2002; Poulin, 1994). If successful, such host manipulation aimed at predation is necessarily fatal for the host. However, not all host manipulation aims at the host's death and some can even enhance host survival, at least temporarily (Dianne et al., 2011; Grosman et al., 2008; Hammerschmidt, Koch, Milinski, Chubb, & Parker, 2009; Weinreich, Benesh, & Milinski, 2013). Nevertheless, any host manipulation likely induces a phenotype that is suboptimal for the host and hence incurs a fitness cost (Maure, Doyon, Thomas, & Brodeur, 2014). Accordingly, an arms race should occur in which the parasite is selected to manipulate its host and the host to resist this host manipulation (Poulin, Brodeur, & Moore, 1994). Indeed, there are some indications that hosts can resist host manipulation. Using different populations of gammarids and their manipulating acanthocephalan parasites, Franceschi et al. (2010) observed that hosts from populations that shared a previous history with the parasites were less manipulated than those from a naïve population, indicating some level of evolved resistance to host manipulation. Similarly, different populations of copepods show slightly different levels of susceptibility to host manipulation by early stage (not yet infective) cestodes *Schistocephalus solidus* (Hafer, 2018). In a virus that manipulates a parasitoid wasp to oviposit in already parasitized hosts facilitating spread of the virus, but reducing wasp fitness, Martinez, Fleury, and Varaldi (2012) observed heritable variation in resistance to host manipulation. In plants, plant defences seem able to partially mitigate the effect of host manipulation by a virus transmitted through white fly vectors (Liu et al., 2017).

To better understand how readily resistance to host manipulation evolves, I experimentally selected the copepod *Macrocyclops albidus* for resistance or susceptibility to host manipulation by the cestode *Schistocephalus solidus*. I used a similar approach and design used recently to investigate the evolvability of host manipulation in *S. solidus* (Hafer‐Hahmann, 2019). *Schistocephalus solidus* is well known to manipulate its first intermediate copepod host to modify its predation susceptibility according to its need; before the parasite becomes infective to its subsequent fish host, it reduces host activity (Benesh, 2010a; Hafer, 2018; Hafer & Benesh, 2015; Hafer & Milinski, 2015; Hammerschmidt et al., 2009) and predation susceptibility (Weinreich et al., 2013). Once the parasite is infective, host manipulation switches to increase host activity (Hammerschmidt et al., 2009; Urdal, Tierney, & Jakobsen, 1995; Wedekind & Milinski, 1996) and hence enhance predation (Wedekind & Milinski, 1996) to facilitate transmission to the next host. Thereby *S. solidus* kills its current host if successful. Under these circumstances, I predict that the host should resist host manipulation.

## 2 METHODS

### 2.1 Hosts

Copepods (*Macrocyclops albidus*) to set up the F0 generation came from a laboratory culture that originated from the brackish lagoon in Neustadt, northern Germany (54°06′49.6″N 10°48′28.0″E). For subsequent generations, I used copepods selected from the previous generation for breeding (see below). Copepods were transferred to individual wells of 24‐well cell culture plates with about 1 ml of water each on the day prior to infection. To measure host manipulation and perform selection, I only used adult male copepods to reduce variability and because, unlike juvenile copepods, they are large enough to easily record their behavior. Adult females could not be used, because they are much larger and less translucent than males making it impossible to reliably check them for infection visually. I checked for dead copepods, cleaned wells if necessary and fed copepods every second to third day with 5 *Artemia* sp. naupili each.

In the last generation (F3), I additionally used copepods from the original laboratory culture (stock) as a control for possible effects of the breeding regime and the artificially high prevalence of *S. solidus*. To set up these tanks, I collected 30 egg‐bearing females from the stock tanks and distributed them to three different tanks one week after I had set up the selected populations (see below) to ensure similar age of the offspring.

### 2.2 Parasites and infection

I used three independent families of *S. solidus* that had been bred in the laboratory for two generations (see Smyth, 1946; Wedekind, 1997 for methods). Their grandparents stemmed from the same population as the copepods (brackish lagoon in Neustadt, northern Germany). *Schistocephalus solidus* eggs can be stored in the fridge (4°C) for some time (Dubinina, 1980), which allowed me to use the same parasite families in each generation. Prior to infection, eggs were incubated for 3 weeks and exposed to light overnight to induce hatching. Infections took place by adding one freshly hatched coracidium to each copepod. During the last generation (F3), 214 (out of 630 exposed) hosts received parasites from one of the other two families, to test for potential adaptations to a specific parasite family.

### 2.3 Selection and breeding

**Figure 1:**
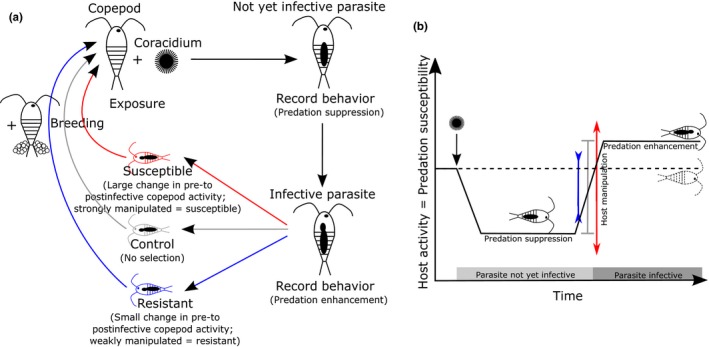
Experimental set up (a) and approach to measuring host manipulation (b) to select hosts for resistance or susceptibility to host manipulation. Please note that only hosts were selected, while parasites always came from the same, unselected, stock in each generation. In each generation, host activity was measured before (predation suppression) and after (predation enhancement) parasites reached infectivity (a). The difference between these two measurements was then considered as host manipulation (b). In the initial generation, copepods were distributed to selection line based on the extent of this host manipulation and then either selected for increased (susceptible) or decreased (resistant) host manipulation. Selected copepods were combined with (unselected) females from the same replicate to breed the next generation (a). Changes in host activity over time (b) are based on Hammerschmidt et al. (2009).

I initially exposed 463 copepods, 159 of which became infected in the initial generation (F0). Subsequently, I set up three different selection lines based on host manipulation (see below; Figure 1a; numbers in brackets represent sample sizes of exposed/infected copepods in each generation and treatment): control (no selection, F1: 98/52, F2: 72/31, F3: 216/77), susceptible (strong host manipulation, F1: 261/126, F2: 216/104, F3: 195/75), and resistant (weak host manipulation, F1: 265/162, F2: 216/95, F3: 219/88). I included unexposed copepods as uninfected control only in the initial (F0, 41 copepods) and the last (F3, 39 copepods in each selection line) generation. In the other generations, I used copepods that were exposed, but remained uninfected to serve as uninfected control copepods to keep the number of copepods manageable. Failed exposure does not seem to significantly affect host behavior in this system (Hammerschmidt et al., 2009). Additionally, I exposed 126 copepods from the stock population to *S. solidus*, 32 of which became infected during F3. In order to obtain a single measure for host manipulation on which I could base selection, I calculated the average activity of each copepod before (day 6–8 postinfection) and after (day 13–15 postinfection) its parasite became infective. I then subtracted these values from each other to obtain the strength of the change in host activity from predation suppression by not yet infective parasites to predation enhancement when parasites became infective (Figure 1b). This resulted in a single value for each copepod for how strongly it is manipulated and is subsequently referred to as “host manipulation.” This also allowed me to obtain a measurement for “host manipulation” in uninfected copepods for comparison even though there of course no host manipulation could take place since there was no parasite. Additionally, using the difference between predation suppression by young and predation enhancement by old parasites, served to avoid selection for different levels of host activity instead of selection for resistance or susceptibility to host manipulation. I included all copepods that were infected and survived until day 15 postinfection in the selection pool. This selection pool was divided into three replicate populations according to the parasite family the copepods had received. These replicates were kept apart throughout the experiment. In the initial generation (F0), one quarter of copepods in each replicate was randomly selected as control line. From the remaining copepods, the third that exhibited the strongest host manipulation was selected to set up the susceptible line, the third that showed the weakest host manipulation the resistant line. If more than ten copepods were available within one selection line and population, I only used the ten most extreme (susceptible and resistant) or ten randomly selected ones (control). Calculating selection differential (i.e., the difference in host manipulation between the entire population and only the selected individuals) showed that there was similar selection both for increased resistance (F0: −0.2971, F2: −0.2471, F3: −0.1785) and susceptibility (F0: 0.2611, F2: 0.2292, F3: 0.2113) throughout the experiment and very little selection in control lines (F0: 0.0042, F2: 0.0447, F3: −0.0001).

All copepods from the same selection line and replicate were combined with twice as many females as males, but at most 15 females. They were maintained in 1 L tanks with a hay sack and aeration and regularly fed with *Paramecium* sp. in the same manner as the stock population. Females always stemmed from the same populations as the males, that is, from the original culture in the F0 generation and from the appropriate selection line and replicate in each subsequent generation. To obtain unmated females, I took juvenile females before they became mature, but after they were recognizable as females and maintained them in small containers with 15 females each in about 150 ml of water until the appropriate replicate population was set up. They were regularly fed with *Paramecium* sp. and any female that started developing eggs during this time was removed.

### 2.4 Behavioral recordings and additional measurements

Copepod behavior was recorded using a device that dropped the plate with copepods by about 3mm in a standardized manner to simulate a predator attack. In order to avoid being attacked by the perceived predator, copepods should reduce their activity following this “attack” (Benesh, 2010a; Hafer & Benesh, 2015; Hafer & Milinski, 2015, 2016; Hammerschmidt et al., 2009). At the beginning of each trial, I placed a plate on the apparatus, let it rest for one minute and then dropped it. The copepod behavior was recorded for 15 min starting just before the drop with a HD‐camera (MHD‐13MG6SH‐D, Mintron, Taiwan). From these video recordings, I extracted one image every 2 s for 90 s in ImageJ (Schneider, Rasband, & Eliceiri, 2012) starting 10 s after the simulated predator attack to exclude the initial reaction (e.g., Hammerschmidt et al., 2009; Benesh, 2010a; Hafer & Milinski, 2015; Hafer‐Hahmann, 2019). To analyse these image sequences, I used a custom‐made python program (available at: https://github.com/ferhah/copepodtracking) that automatically recorded copepod position (Hafer, 2018; Hafer‐Hahmann, 2019). To exclude random noise, a copepod was only considered moving if it moved by at least 5 pixel (about one copepod length). These data were used to calculate the proportion of time each copepod spent moving during the first 90 s following the simulated predator attack. Behavioral recordings took place three times before parasites became infective when they should suppress predation (day 6, 7, and 8 postinfection) and three times after parasites had become infective when they should enhance predation (day 13, 14, and 15 postinfection). On day 8 postinfection and after that day's behavioral recordings, I checked copepods for parasite infection by placing them on a microscope slide with just enough water to ensure their survival. If done quickly, this does not seem to adversely affect the copepod (personal observation) and is a routine procedure in this system (Benesh, 2010b; Benesh & Hafer, 2012; Hafer‐Hahmann, 2019; Hammerschmidt et al., 2009). To investigate whether selection on the host for resistance or susceptibility to host manipulation would result in changes in other parasite traits, I additionally recorded parasite development and size. To record development, the presence or absence of a cercomer was recorded on day 8 postinfection while checking copepods for infection. The cercomer presents a good indicator for parasite development; however, its development precedes infectivity by several days, so even parasites with a cercomer 8 days postinfection are not yet infective to fish (Benesh, 2010a; Benesh & Hafer, 2012). On day 15 postinfection, each copepod was again placed on a microscope slide and photographed to measure parasite size. To do so, I measured parasite size from these photos by outlining each parasite's shape without the cercomer and measuring the area within this shape (Wedekind, Christen, Schärer, & Treichel, 2000) in ImageJ (Schneider et al., 2012). At this time, all but one out of about 1,000 parasites that successfully infected copepods in this experiment had a cercomer.

### 2.5 Statistical analysis

All statistical analysis were conducted in R, version 3.5.1 (R Development Core Team, 2016). Copepods in F0 were randomly assigned to a selection line for statistical analysis, which did not necessarily correspond to the selection line, which they were selected for.

To analyse copepod behavior (host manipulation and host activity), I excluded all copepods that had died or been lost during the experiment or in which exposure had not resulted in an infection, except during F1 and F2 when exposed but uninfected copepods served as uninfected controls. To analyse infection success, parasite development, and parasite size, I used all copepods for which the relevant information could be obtained.

To each response variable, I applied general (host manipulation, host activity, and parasite size) or generalized linear mixed models with a binomial error family (infection success and development) from the lme4 package (Bates, Maechler, Bolker, & Walker, 2015). Replicate was included as random variable and generation, selection line, and their interaction as fixed factors. To analyse host manipulation (i.e., the difference between host activity within each copepod before and after its parasite became infective) and host activity (i.e., copepod activity before or after parasites became infective), I additionally included infection status of the copepod and all its statistical interactions with the fixed factors. The model for host activity included two additional factors, a fixed factor parasite stage (i.e., predation suppression by not yet infective parasites versus predation enhancement by infective parasites) and all its interactions with the other fixed factors and a random factor copepod identity to account for the fact that each copepod was measured twice, that is, before and after its parasite reached infectivity. To test whether the statistical assumptions were met, I checked the distribution of the residuals from the best model for each response variable (see below) for normality and homogeneity. I used two sample Kolmogorov–Smirnov tests comparing the distribution of the residuals to a random normal distribution with the same mean and sd as the residuals in question using 10,000,000 observations and the Bartlett test to test the homogeneity of variance. The results of these tests confirmed that the assumptions were met in each case (Host manipulation: Normality: D = 0.027, *p* = 0.204, homogeneity: K2 = 1.717, *df* = 2, *p* = 0.424; host activity: normality: D = 0.010, *p* = 0.944, homogeneity: K2 = 5.397, *df* = 2, *p* = 0.067; parasite size: normality: D = 0.041, *p* = 0.254, homogeneity: K2 = 1.638, *df* = 2, *p* = 0.441).

For each analysis, I compared each model to a less complicated model using AIC. To obtain p values for this comparison, I applied likelihood ratio tests. A factor was considered to have a significant effect if the model containing it explained the data significantly better than a less complicated one without that factor. If I found an effect of selection line (or any interaction involving selection line), I conducted a post hoc tests using Tukey corrections for multiple testing on the best models containing all effects of interest using emmeans (Lenth, 2019). I conducted pairwise comparisons between selection lines overall and within each level of infection, parasite stage, and generation if their interactions with selection line were at least a nonsignificant trend.

To test for a potential effect of the breeding procedure on host manipulation and activity, I fitted general linear mixed models to data from F3 only. Using log likelihood ration tests, I compared a model that contained whether or not a copepod belonged to the stock population, infection, and parasite stage (activity only). This was followed up by a post hoc test using emmeans with Tukey corrections (Lenth, 2019) on the same model used previously, but containing treatment instead of stock as a fixed factor to investigate with which selection line any differences occurred.

To confirm repeatability of the measurements for each copepod during each parasite stage, I estimated repeatability from generalized mixed effect models with maximum likelihood fitting using rpt (Stoffel, Nakagawa, & Schielzeth, 2017). The model used contained the random effect copepod identity, which was also used as grouping factor. Copepod behavior was repeatable between days (*R* ± *SE*; day 6–8: *R* = 0.729 ± 0.010, *p* < 0.001; day 13–15: *R* = 0.638 ± 0.012, *p* < 0.001).

To keep the results easier to read, only the most relevant *p* values (e.g., those focusing on differences between selection lines) are presented in the results. For more details on the statistical outputs of the models, please refer to Tables 1, 2, 3, 4.

**Table 1:** General linear mixed models to analyse copepod behavior in response to selection for susceptibility or resistance to host manipulation

Factor	*df*	AIC	χ^2^	*p*
Host manipulation (AIC: −620, *df*: 3)				
+Generation	4,1	−654	36.28	**<0.0001**
+Infection	5,1	−804	152.15	**<0.0001**
+Selection line	7,2	−808	7.38	**0.0250**
+Infection: Selection line	9,2	−809	5.22	0.0736
+Generation: Infection	10,1	−808	0.97	0.3243
+Generation: Selection line	12,2	−808	4.40	0.1111
+Generation: Infection: Selection line	14,2	−804	0.18	0.9130
Host activity (AIC: −886, *df*: 4)				
+Parasite stage (predation suppression vs. predation enhancement)	5,1	−1611	726.86	**<0.0001**
+Generation	6,1	−1697	88.26	**<0.0001**
+Generation: Parasite stage	7,1	−1723	27.74	**<0.0001**
+Infection	8,1	−2312	591.58	**<0.0001**
+Infection: Generation	9,1	−2312	1.73	0.1884
+Infection: Parasite stage	10,1	−2428	117.19	**<0.0001**
+Selection line	12,2	−2481	57.10	**<0.0001**
+Selection line: Parasite stage	14,2	−2482	5.32	0.0700
+Selection line: Generation	16,2	−2478	0.19	0.9109
+Selection line: Infection	18,2	−2474	0.20	0.9090
+Selection line: Infection: Generation	20,2	−2476	5.37	0.0683
+Selection line: Infection: Parasite stage	22,2	−2477	5.17	0.0752
+Selection line: Infection: Parasite stage: Generation	25,3	−2474	3.37	0.3381

Note

Initial model for host manipulation: response ~1 + (1|replicate). Initial model for host activity: response ~1 + (1|replicate) + (1|copepod identity). *N*: Host manipulation: 1,517 copepods in three replicates; host activity: 3,034 observations on 1,517 copepods in three replicates. Significant *p* values are highlighted in bold.

**Table 2:** Summary of the general linear models to determine the effect of selection line on host manipulation and host activity containing all fixed effects and significant interactions

	Host manipulation	Host activity		
	Variance ± *SD*	Variance ± *SD*		
Random effects				
Id (intercept)		0.0031 ± 0.0558		
Replicate (intercept)	0.0005 ± 0.0217	0.0004 ± 0.0196		
Residual	0.0340 ± 0.1845	0.0231 ± 0.1520		

	Host manipulation		Host activity	
	Estimate ± *SE*	*t*	Estimate ± *SE*	*t*
Fixed effects				
(Intercept)	0.154 ± 0.019	7.93	0.514 ± 0.016	31.7
Parasite stage			0.160 ± 0.013	12.2
Generation	−0.029 ± 0.005	−5.69	−0.022 ± 0.004	−5.09
Generation: parasite stage			−0.031 ± 0.006	−5.34
Infection	0.124 ± 0.010	12.7	−0.225 ± 0.008	−26.7
Infection: parasite stage			0.125 ± 0.011	11.1
Selection line (high)	0.018 ± 0.013	1.43	0.031 ± 0.008	3.77
Selection line (low)	−0.011 ± 0.013	−0.84	−0.021 ± 0.008	−2.59

Note

Comparisons are with copepods from the control line infected by not yet infective (host activity only) parasites.

**Table 3:** Post hoc tests for selection on hosts for susceptibility or resistance to host manipulation

Comparison	A: Host manipulation		B: Predation suppression								C: Predation enhancement							
			F0		F1		F2		F3		F0		F1		F2		F3	
	*t*	*p*	*z*	*p*	*z*	*p*	*z*	*p*	*z*	*p*	*z*	*p*	*z*	*p*	*z*	*p*	*z*	*p*
Uninfected: control ‐ high	−0.92	0.627	−0.60	0.818	−0.54	0.851	−0.81	0.697	−0.61	0.815	−0.85	0.671	−1.01	0.571	−1.28	0.404	−1.00	0.577
Uninfected: control ‐ low	−0.82	0.689	0.19	0.979	2.60	**0.025**	0.64	0.800	−0.35	0.936	−0.14	0.990	2.01	0.109	0.02	1	−0.85	0.669
Uninfected: high ‐ low	0.12	0.992	0.78	0.713	4.21	**<0.001**	1.98	0.118	0.28	0.959	0.72	0.754	4.04	**<0.001**	1.80	0.171	0.16	0.985
Infected: control ‐ high	−1.17	0.471	0.09	0.996	−1.76	0.1837	0.55	0.844	−3.00	**0.008**	−0.50	0.873	−2.41	**0.042**	0.02	1	−3.65	**<0.001**
Infected: control ‐ low	1.74	0.191	−1.15	0.484	1.16	0.4802	1.23	0.435	−1.33	0.380	0.26	0.964	2.90	**0.011**	2.61	**0.024**	0.41	0.910
Infected: high ‐ low	3.38	**0.002**	−1.23	0.433	3.83	**<0.001**	0.97	0.595	1.79	0.173	0.72	0.755	6.95	**<0.001**	3.70	**<0.001**	4.26	**<0.001**

Note

Comparisons between selection lines were conducted within each level of interactions that showed a nonsignificant trend. Significant p values are highlighted in bold.

**Table 4:** General and generalized linear mixed models to analyse changes in parasite traits during selection on hosts for susceptibility or resistance to host manipulation

Factor	*df*	AIC	χ^2^	*p*
(a) Infection success (AIC: 2,867, *df*: 2)				
+Generation	3,1	2,867	1.27	0.260
+Selection line	5,2	2,870	1.13	0.569
+Generation: Selection line	7,2	2,872	2.67	0.263
(b) Parasite development (AIC: 924, *df*: 2)				
+Generation	3,1	924	2.32	0.128
+Selection line	5,2	922	5.64	0.060
+Generation: Selection line	7,2	926	0.02	0.989
(C) Parasite size (AIC: 3,489, *df*: 3)				
+Generation	4,1	3,491	0.27	0.601
+Selection line	6,2	3,495	0.20	0.904
+Generation: Selection line	8,2	3,495	3.47	0.177

Notes

Initial model: response ~ 1 + (1|replicate). *N*: Infection success: 1,896 copepods in three replicates; parasite development: 718 copepods in three replicates; parasite size: 602 copepods in three replicates. Please note that the nonsignificant trend (*p* = 0.060) for an effect of selection line on parasite development is an artifact of the random assignment of each copepod to a selection line for statistical modeling during the initial generation (Effect of selection line without F0: χ^2^ = 1.46, *p* = 0.482).

## 3 RESULTS

**Figure 2:**
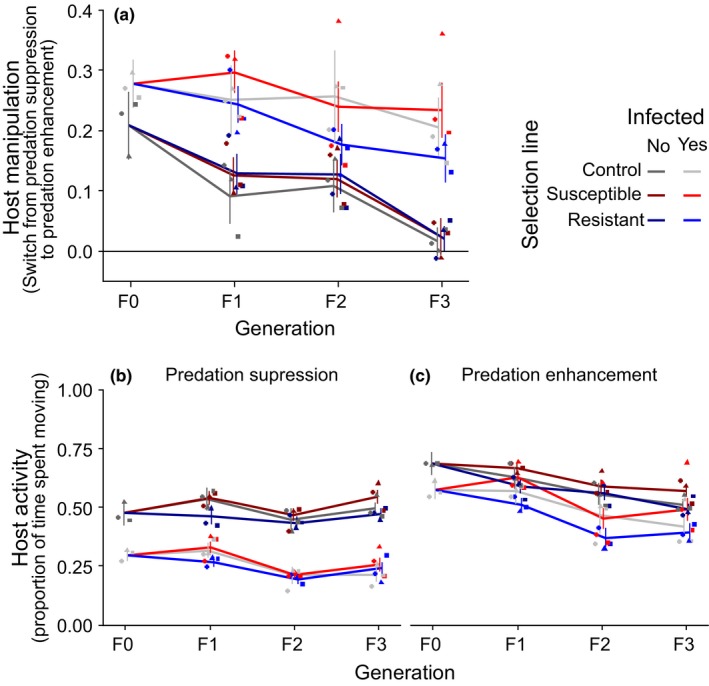
Resistance to host manipulation over generations. (a) host manipulation, (b) predation suppression, (c) predation enhancement. *N* (uninfected/ infected): F0: 35/146, F1: control: 34/51, susceptible: 102/122, resistant: 81/154, F2: control: 35/29, susceptible: 99/102, resistant: 110/92, F3: control: 33/73, susceptible: 32/72, resistant: 34/82. Different symbols indicate different replicate populations each corresponding to a different parasite family during selection. Error bars represent 95% confidence intervals

As expected, infected copepods showed higher levels of “host manipulation” (i.e., the change in host activity as the parasite became infective and switched from predation suppression to predation enhancement or the change in host activity during the respective time without parasite in uninfected copepods) than uninfected ones (*p* < 0.0001, Figure 2a, Table 1). Somewhat surprisingly, “host manipulation” was not zero in uninfected copepods as would be expected if their behavior did not change over time. It is possible that copepods became habituated thereby remaining increasingly more active after the simulated predator attack even if they were not infected. Overall host manipulation decreased between generations (*p* < 0.0001, Figure 2a, Table 1); however, since this occurred irrespective of infection and selection line (*p* > 0.1, Figure 2a, Table 1), this decrease is unlikely to be connected to actual host manipulation.

Selection resulted in altered behavior in accordance with selection regime (*p* = 0.0250, Table 1) by increasing host manipulation in susceptible and decreasing it in resistant lines (*t* = 2.70, *p* = 0.0191) with controls being intermediate between susceptible (*t* = −1.42, *p* = 0.3287) and resistant selection lines (*t* = 0.84, *p* = 0.6780) and not significantly different from either. Interestingly, the interaction with infection was only a nonsignificant trend (*p* = 0.0736), indicating that the effect of selection line might not have been restricted to infected copepods, albeit post hoc tests following up this trend found that susceptible copepods were only significantly more active than resistant ones if they were infected (infected copepods: *p* = 0.0022; uninfected copepods: *p* > 0.9, Figure 2a, Table 3a).

To better understand whether these differences in host manipulation were due to increased susceptibility or resistance to predation suppression by not yet infective parasites or to predation enhancement by infective parasites, I looked at host activity (i.e., proportion of time spent moving after a simulated predator attack) during both parasite stages. Not surprisingly, activity was lower during predation suppression (*p* < 0.0001, Figure 2b, c, Table 1) and was significantly reduced by infection (*p* < 0.0001, Figure 2b, c, Table 1). As for host manipulation, selection line had a significant effect on host activity (*p* < 0.0001, Figure 2b, c, Table 1); susceptible lines were more active than resistant ones (*z* = 7.75, *p* < 0.0001) with control lines intermediate and significantly different from both susceptible (*z* = −4.00, *p* = 0.0002) and resistant lines (*z* = 2.39, *p* = 0.0449). According to selection regime, susceptible copepods should have become less active during predation suppression and more active during predation enhancement, but only if infected. Yet, there was only a nonsignificant trend for the effect of selection line to depend on parasite stage (*p* = 0.0700, Figure 2b, c, Table 1) and none that the effect of selection line was restricted to infected copepods (*p* > 0.9, Figure 2b, c, Table 1). Hence, not all differences between selection lines could be due to changes in how copepods reacted to host manipulation. There were, however, nonsignificant trends that the effect of infection on the effect of selection line varied depending on parasite stage (*p* = 0.0752, Figure 2b, c, Table 1) and generation (*p* = 0.0683, Figure 2b, c, Table 1). These trends seem to have been driven by susceptible copepods being more active than resistant ones even during predation suppression or if they were not infected during the first generation (*p* < 0.0003, Table 3b, c, Figure 2b, c). After the first generation, differences between selection lines in uninfected copepods and during predation suppression diminished (*p* < 0.1, Table 3b, c, Figure 2b, c). During predation enhancement, susceptible copepods were more active than susceptible ones and this differences persisted over the course of the experiment (*p* < 0.0007, Table 3b, c, Figure 2b, c).

Comparisons with copepods from the stock populations revealed no clear effect of the breeding procedure on copepod behavior (Host manipulation: AIC: −214 vs. −216, χ^2^ = 3.76, *p* = 0.0523; Host activity: AIC: −707 vs. −707, χ^2^ = 1.99, *p* = 0.1585); post hoc tests following up on the nonsignificant trend with regard to host manipulation confirmed that differences with the stock population were not consistent between treatments, but only occurred when comparing copepods from the stock population to those from the resistant selection lines (*t* = −2.93, *p* = 0.0187), but not when compared to control or susceptible lines (*p* < 0.5). There was no specificity of resistance or susceptibility to a certain parasite family (AIC: −83 vs. −85, χ^2^_6,1_ = 0.02, *p* = 0.902). Analysis of other parasite traits revealed no significant changes due to selection for susceptibility or resistance to host manipulation (*p* > 0.05, see Table 4).

## 4 DISCUSSION

The copepod host of *S. solidus* was unable to quickly evolve resistance or susceptibility to host manipulation. By contrast, host manipulation changed within a few generation in a similar experiment in which only the parasite was under selection (Hafer‐Hahmann, 2019). Instead of evolving actual susceptibility to host manipulation, susceptible copepods became more active than resistant ones, irrespective of infection and parasite stage and hence not because of altered susceptibility or resistance to host manipulation. In infected copepods, this altered level of activity was decreased by not yet infective parasites and increased by infective parasites. Manipulation during predation suppression seems to have been more efficient in lowering host activity irrespectively of baseline levels of host activity than predation enhancement was in increasing host activity. This resulted in overall differences in host manipulation on which selection was based. That the host alters its baseline behavior rather than actually resisting host manipulation and thereby possibly modifying its interaction with the parasite also fits well with the fact that selection in this experiment had no effect on any of the other parasite traits measured in this study. In nature such a response could nevertheless mitigate some of the phenotype created by host manipulation, but at least in this system, it is unclear to which extent that would ultimately benefit the host, since in copepods prevalence of *S. solidus* in nature seems to be usually low (Hanzelová & Gerdeaux, 2003; Pasternak, Huntingford, & Crompton, 1995; Zander, Groenewold, & Strohbach, 1994). It fits well, however, with previous speculations that rather than actually resisting host manipulation, hosts could alter their baseline behavior to counter and accommodate host manipulation by a very prevalent and co‐evolved parasite, but resulting in a suboptimal phenotype in the absence of this parasite (Hafer, 2016; Read & Braithwaite, 2012; Weinersmith & Earley, 2016). With time, differences between uninfected copepods from different lines during predation suppression by not yet infective parasites decreased. Therefore, it is plausible that given sufficient time hosts would eventually evolve actual resistance or susceptibility to host manipulation.

That the host seems better able to mitigate the altered behavior during predation enhancement than during predation suppression is surprising in light of a recent study in the same system that investigated differences between populations (Hafer, 2018). There the level of predation suppression depended only on host, but not parasite population. By contrast, predation enhancement was only determined by parasite population (Hafer, 2018). It could be that variation for resistance to predation suppression exists between populations but is very limited within each population, and hence, selection has little to act on. From the host's perspective, if it is unable to evolve resistance to both predation suppression and predation enhancement equally well, favouring resistance to predation enhancement might be the better strategy, since the consequences if the parasite succeeds—predation—are much more severe.

## AUTHORS CONTRIBUTION

I am the sole author of this manuscript.

## Data Availability

Data are available in Dryad (https://doi.org/10.5061/dryad.cb2f70h).
